# Layer-by-layer assembly: advancing skin repair, one layer at a time

**DOI:** 10.1039/d4ra08115c

**Published:** 2025-04-29

**Authors:** Elias Hasan, Christopher J. Lewis, Joel Giron Hernandez, Piergiorgio Gentile, Ana M. Ferreira

**Affiliations:** a School of Engineering, Faculty of Science, Agriculture & Engineering, Newcastle University Newcastle upon Tyne UK e.a.h.h.hasan2@ncl.ac.uk Piergiorgio.gentile@ncl.ac.uk ana.ferreira-duarte@ncl.ac.uk; b Northern Regional Burn Centre, Royal Victoria Infirmary Newcastle upon Tyne UK christopher.lewis10@nhs.net; c Applied Sciences, Northumbria University Newcastle upon Tyne UK jo-el.l.g.hernandez@northumbria.ac.uk

## Abstract

Skin wound management remains a critical global healthcare challenge, with annual costs exceeding £30 billion. Traditional treatments like autografts face limitations in cost, availability, and recovery times. This review explores spray-assisted Layer-by-Layer (LbL) technology as a transformative approach for wound healing, emphasising its ability to deposit natural- and synthetic-polyelectrolytes such as chitosan, alginate, hyaluronic acid, and collagen into nanoscale coatings. These biocompatible multilayers integrate therapeutic agents to accelerate healing, reduce infections, and mimic native extracellular matrix structures. The work highlights emerging spray device innovations that optimise spray parameters to enhance cell viability, coverage, and clinical outcomes. While LbL techniques demonstrate versatility across substrates and scalability *via* immersion, spray, and microfluidic methods, challenges persist in manufacturing uniformity and clinical translation. The review underscores the urgent need for clinical trials to validate Lbl-based coatings in real-world settings and addresses gaps in portable, sustainable device development. By bridging advanced materials science with clinical practice, spray-assisted LbL technology offers a roadmap to overcome current wound care limitations, prioritising biocompatibility, cost-efficiency, and improved patient safety in regenerative medicine.

## Abstract 2

Skin wound management remains a critical global healthcare challenge, spray-assisted LbL technology offers an alternative to overcome current limitations.

## Graphical abstract

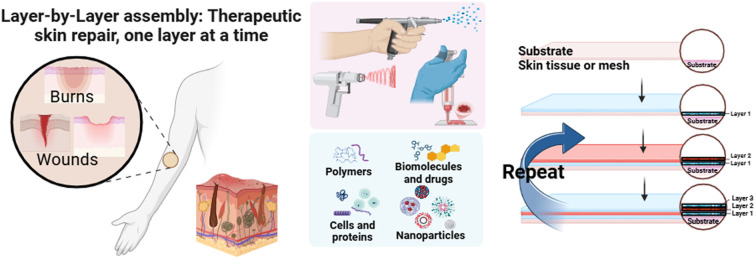

## 1. Introduction

In recent years, skin wound management has seen significant advancements, particularly in the development of cellular approaches and clinical technologies to enhance skin tissue regeneration. Human skin is the body's largest organ, covering approximately 1.6–2.0 m^2.^^1^ It serves as a boundary that protects the body from external threats, such as pathogenic bacteria and environmental factors. Due to its protective role, the skin is susceptible to injuries resulting in wounds or trauma. A wound refers to any disruption or damage to the skin's surface, while trauma denotes a severe, often life-threatening injury to the body.^2^ The human body can generate healing factors, including specific proteins and cells (*e.g.*, epidermal growth factor (EGF), Interleukin-1 (IL-1), collagen type I, fibroblasts, keratinocytes, and immune cells), which aid in the wound healing process (WHP). This natural healing ability is more effective for small and superficial wounds than for wounds larger than 4 cm^2^. Therefore, medical assistance is crucial to effectively support the healing process. Autografts are currently regarded as the gold standard for mimicking natural skin composites to accelerate wound healing in skin tissue engineering, despite the associated complications, such as infections, burning sensation, compromised muscle strength, impaired wound healing, and numbness.^3^ Therefore, there is a significant need for alternatives capable of speeding up the healing process, as the high cost of production and wound management continues to rise.^4^ Indeed, the World Health Organization (WHO) reports that the current global cost for wound management is between £10 and £30 billion, excluding the size, type of wound, and existing wound therapies.^5^

While the economic burden of skin wound management is substantial, a comprehensive understanding of the biological and clinical background of wounds and their healing processes is crucial for developing effective treatments. Wounds can be classified into various types, such as bacteria-infected wounds and diabetic wounds, each with unique recovery challenges and treatment strategies.^6^ Key aspects, such as the wound depth, risk factors, and severity, enable clinicians to determine the appropriate wound healing treatment.^7^ Depending on the severity, current skin treatment in clinics may range from simple interventions (*e.g.*, cleaning, topical treatment with antibiotics, *etc.*) to more advanced treatments, including wound dressing and surgical interventions, such as skin grafts for extensive skin loss. Due to the limited availability of autografts, alternative approaches to wound healing are essential.^8^

Promising techniques to accelerate wound healing include cell therapy and regenerative medicine.^9^ However, these methods have limitations, including the need for a sterile environment, lengthy cell expansion times, and high costs.^10^ Emerging technologies, such as portable and non-portable wound care devices, offer new ways to deliver wound care management, providing benefits in improving patient outcomes and cost reduction.^11^ Particularly, those leveraging nanoscale-based strategies have shown promise in accelerating wound healing and improving outcomes. For instance, the spray-assisted layer-by-layer assembly technique has been utilised to deposit polyelectrolyte multilayer films on hyaluronic acid scaffolds, promoting cell adhesion and regeneration of the epidermal barrier functions of the skin.^12^

However, several challenges and gaps remain in both clinical and research domains. Clinically, the complexity of skin architecture, which includes multiple layers with distinct cellular compositions, poses a significant challenge in replicating natural skin structures. Additionally, ensuring efficient vascularisation to provide adequate blood supply to the regenerating tissue is critical but difficult to achieve.^13^ Research gaps include the need for more advanced biomaterials that can mimic the natural extracellular matrix and support long-term tissue integration.^14^ Moreover, the scalability and cost of producing these advanced treatments remain significant barriers to widespread clinical adoption.

This review aims to comprehensively analyse the latest advancements in layer-by-layer assembly (LbL) and clinical technologies in skin regeneration, highlighting the potential of integrating these approaches to advance skin tissue regeneration. This method is noted for its versatility in incorporating biomolecules, therapeutic drugs, antimicrobials, and other agents to improve wound care management. By addressing the existing challenges and gaps, we can pave the way for more effective and accessible treatments for various types of wounds.

## 2. Layer-by-layer assembly overview

LbL creates ultrathin layers on substrates by alternately depositing oppositely charged species, resulting in a multilayered structure. The technique is highly versatile in regenerative medicine approaches, as it facilitates the incorporation of small bioactive molecules and biological agents at the nanoscale, enhancing the therapeutic properties of the assembled films or coatings.^15^ Furthermore, the LbL strategy can exploit a wide range of natural and synthetic polyelectrolytes (PEs), which can be tailored for a controlled release by tuning parameters such as pH level, number of layers, polyelectrolyte, salt concentration and temperature.^16^

**Fig. 1:**
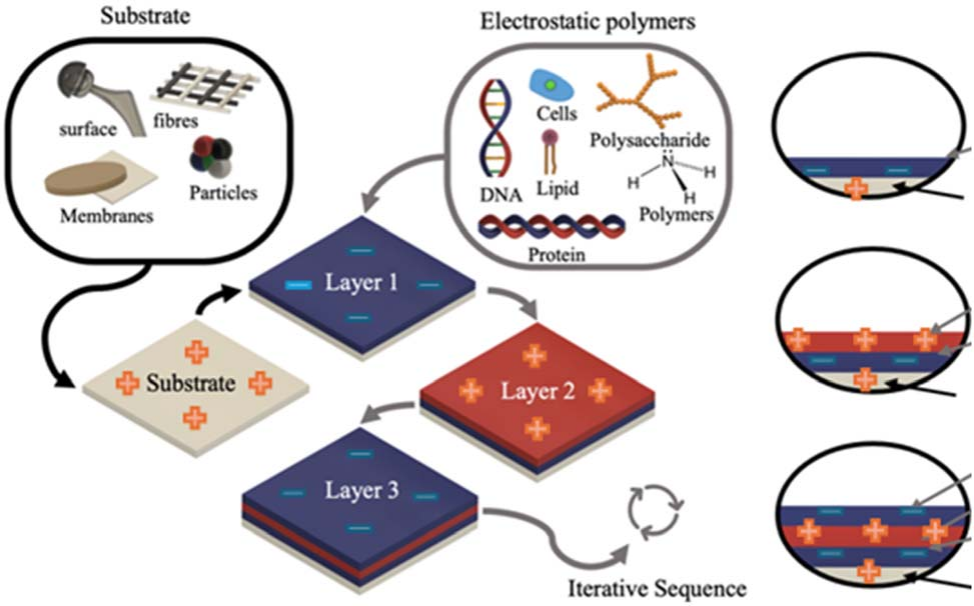
Layer-by-Layer (LbL) assembly of polyelectrolytes (PEs) on a charged substrate (in grey, indicated by black arrows) is used to construct multiple layers (blue and red, process indicated by grey arrows). This process allows for a variety of oppositely charged substrates with materials as PEs, to be used in fabrication.

The PEs can be synthetic or biological materials (*e.g.*, lipids, proteins, DNA, cells, and polysaccharides) that contain positive or negatively charged units. For instance, polysaccharides that carry a negative electrostatic charge, such as alginate and pectin, have carboxyl groups that lose a proton from their sugar units. This process forms negatively charged carboxylate ions (COO^−^). The degree of negative charge varies depending on the type of polysaccharide and the pH of the solution. Polysaccharides, such as chitosan, are primarily composed of positively charged or protonated units. For instance, amino groups (NH_2_) become protonated to form NH_3_^+^ at physiological pH or slightly acidic conditions, below the isoelectric point. Similarly, lipids hold a negative electrostatic charge due to the presence of the phosphate group.^17^ A protein can have both negative and positive charges according to its isoelectric point, and changes in physiological pH. DNA has a highly negative charge due to the presence of phosphate.^18^ Therefore, the LbL assembly method exploits electrostatic interactions of oppositely charged polyelectrolytes (PEs)^19^ or polymers to build up multiple layers at nanoscale precision onto a charged substrate, enabling the control of multilayers' thickness and possible weight management.^19^ Thus, the LbL process involves a sequential deposition of two or more materials that are oppositely charged^20,21^ to interact physically, leading to either weak or strong electrostatic interactions depending on the materials' chemical nature (Fig. 1). A weak ionic bond is affected by the pH level (with a dissociation constant varying between 2 and 10). In contrast, the strong PEs' electrostatic interaction is more stable and not easily affected by pH level changes. Moreover, the counterions are critical factors that determine the ionic strength between PE multilayers to ensure counterbalance and a well-defined LbL structure.^22^ Similarly, combining strong/weak PE improves the stability of the multilayer, whereas weak/weak PE reduces stability and may facilitate the release of the assembled layers. Moreover, weak PEs are capable of linking with neutral polymers; however, the pH level and hydrogen bond affect the bond strength between the PE and polymers, as the ion pairs formation increases the degree of electrostatic interaction.^23^

The substrate's surface charge and physical–chemical properties enable the deposition and further assembly of the oppositely charged PEs electrostatically, influencing the process and physical–chemical properties of the engineered films. The substrate's charge guides the initial sequence of layer deposition and therefore the stability and properties of the resulting multilayered film in the polyelectrolyte multilayer (PEM) formation process.^24^

**Fig. 2:**
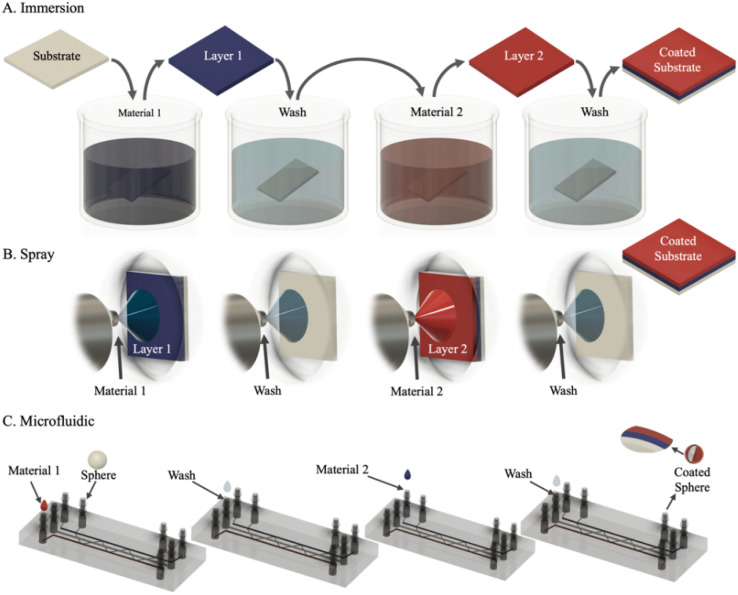
Illustration of the three commonly used Layer-by-layer methods in biomedical technologies: (A) immersion or dipping, (B) spray and (C) microfluidic, showing the iterative deposition of material 1, washing step and material onto substrates, being in a microfluidic system, micro or nanoparticles passing through the PEs solutions.

Fig. 1 illustrates the roles of the substrate and the assembly process of three layers (layers 1–3). Assuming the substrate is positively charged, the deposition follows with negatively (blue), positively (red), and negatively (blue) charged layers, and so on. Depending on the substrate geometry and size, the nature and desired properties for the target application, the LbL method can change. Indeed, Caruso *et al.*^24,25^ have presented comprehensive research about the use of different methods to achieve multilayer coatings through techniques such as immersion or dipping, spraying, spinning, electromagnetic and microfluidic.^26^ These five distinct routes of assembly, each of which offers material and processing advantages for assembling layer-by-layer films, have been described in detail by Richardson *et al.*^25^ where the choice of materials allows for responsive and functional thin films to be engineered for various applications. Further, Fig. 2 displays immersion, spray, and microfluidic techniques, which are the most used methods of LbL assembly in building blocks. These are presented to illustrate the iterative sequence to build up PEMs based on the intended application. For example, the immersion method is one of the most used techniques for LbL assembly due to its simplicity, cost-effectiveness, and high stability on coated substrates. While it allows for precise deposition of layers, it can be time-consuming and may not be suitable for rapid production.^27,28^ In contrast, the spray LbL method is preferred for large-area coatings, allowing for fast deposition and high scalability while providing reliable results at a lower cost. However, it may present challenges in achieving uniformity and may lead to material loss. This technique is particularly suitable for applications involving drug-loaded biopolymers and nanoparticle-functionalised surfaces.^29^

Microfluidic-assisted LbL assembly is a specialised technique designed for micromodels to enhance functionality, such as mechanical properties or adhesion of a micro- or nano-sized substrate.^30^ While it offers precise control over layer thickness, making it highly effective for drug delivery and biomedical applications, this procedure is time-consuming and labour-intensive. Additionally, it requires a complex setup and may be challenging to scale. For instance, commonly used PEs in this context are DNA, protein-based molecules, and growth factors, all of which exhibit significant compatibility with microfluidic channels, thus enhancing their effectiveness in such applications.

The LbL assembly application is versatile and offers advantages to be incorporated into different industrial and technological settings^25^ from large-scale to nano-structure applications.^31^ The fabrication methods of LbL films enable to addition of combinatorial properties through composites, structure, and functions in a simple, robust and versatile yet reproducible approach.^32^ These physicochemical properties are commonly characterisation in terms of their surface energy, chemical composition and bonds, and physical and morphological properties, among others, as comprehensively described elsewhere^24^ to monitor LbL assembly.

### 2.1. Therapeutic polyelectrolytes in skin wound healing

Nature-based biopolymers derived from living organisms, such as plants and animals, are used as PEs in LbL applications due to their biocompatibility, biodegradability and non-cytotoxicity.^26^Table 1 presents how different of these naturally derived materials have been in skin wound healing and medical devices or therapeutic approaches (cleared under the U.S. Food and Drug Administration (FDA) 510(k) classification as safe and effective as a legally marketed device^33,34^) due to their chemical and biological properties. For instance, among the polysaccharides, chitosan (CHI) helps moisturise wounds, prevents them from drying, and provides antimicrobial properties.^35,36^ Similarly, alginate (ALG) assists in absorbing exudates to form a gel, maintaining a moist environment, minimising bacterial infections and promoting re-epithelialisation and granulation for tissue formation.^37^ Another example is pectin, a versatile biopolymer that plays a crucial role in polyelectrolyte LbL assembly due to its strong electrostatic interactions, low toxicity, and ability to enhance the mechanical properties of the resulting structures. Its importance is further highlighted by its capacity to form stable complexes with oppositely charged macromolecules, making it an excellent candidate for creating robust and biocompatible materials.^38^ Pectin is employed in the biomedical and pharmaceutical sectors for its cytocompatibility, which is versatile for various applications such as drug delivery, wound healing, and tissue engineering. Pectin creates a stable, effective topical treatment employing an advantageous therapeutic approach.^39^ These examples highlight the advantages of different polysaccharides as PEs in wound care applications.

**Table 1:** Summary of widely used naturally-derived materials used as polyelectrolytes and their current applications in skin repair

Polyelectrolyte		Classification	Biological properties	Medical device 510(k)	Ref
Polycations	Chitosan	Weak	Promotes tissue organisation, antibacterial agent, homeostatic (blood clotting), enhances mechanical stability	Foshan UMT LTD KA01 chitosan (wound dressing)	42
	Collagen type I	Weak	Biocompatible, low immunogenicity, main ECM structural protein, biodegradable	CoMatryx (bovine) (wound dressing), medifil gel (bovine) (suspension), hyCure (bovine) (powder), integra flowable hydrated granules	43
Polyanions	Alginate	Weak	Reduces healing-related infections with pathogenic microorganisms, low toxicity	LUOFUCON@ PHMB alginate dressing and antibacterial alginate (wound dressing)	23
	Hyaluronic acid	Weak	Biodegradable, anti-inflammatory, mucoadhesive, enhances viscoelastic properties	Virchow Biotech Pvt Ltd hyaluronic acid topical wound cream 0.2%w/w	43 and 44
	Heparin	Strong	Promotes tissue adhesion and reduces scar tissue formation. Enhances epidermal regeneration	Heparin sodium injections (anticoagulant)	
	Methylglyoxal	Weak	Accelerates dermal repair and epithelialisation, reduces healing-related infections with pathogenic microorganisms	Medihoney® dressing, Surgihoney® dressing	36, 45 and 46
	Pectin	Strong	Low toxicity, enhances mechanical properties	Innovative technologies LTD Alginate/Pectin wound dressing	47

As a protein and natural polymer, collagen Type I, a key extracellular matrix component, plays a crucial role in LbL for maintaining the structural integrity of PEs multilayers. Its biocompatibility, biodegradable properties, and low immunogenicity make it ideal for diverse wound healing products. Collagen Type I enhances the mechanical stability and biological functionality of LbL coatings, making them effective for wound repair and tissue regeneration. For instance, Startagraft is a bioengineering skin substitute that serves as a structural framework that mimics the natural dermal layer of skin, which is essential for supporting cell adhesion and proliferation during the healing process, largely due to the presence of collagen Type I. Its biocompatibility and biodegradability minimise the immune response, thereby improving the graft's integration and efficacy in treating severe burns and skin defects, ultimately eliminating the need for donor skin harvesting.^40^ Then, hyaluronic acid (HA) plays a significant role in the WHP, promoting cell migration and angiogenesis through cell receptors across all healing processes despite its poor mechanical properties. Heparin (HEP) promotes capillary circulation and reduces healing time.^41^

Another example of naturally derived compounds with advantageous properties in the treatment of skin is methylglyoxal (MGO), a compound found in manuka honey, which has gained interest as a new antibacterial biomaterial in the last decade. MGO is known for its antibacterial properties and has shown significant potential in managing wounds. It effectively interacts with various types of wounds, including excisions, incisions, and burns, especially those with light to moderate exudation.^36^

### 2.2. Multilayered coatings with antibacterial properties

Bacteria are well known for disrupting the tissue microenvironment by rapidly colonising wounds and burn areas, leading to infections and further hindering the WHP. The most predominant bacteria in wound injuries such as the Gram-positive *Streptococcus pneumoniae* (*S. pneumoniae*) and *Staphylococcus aureus* (*S. aureus*), as well as the Gram-negative *Escherichia coli* (*E. coli*).^46^ Indeed, wound infections can occur during the healing process, and antimicrobial bioactive wound dressing can be more effective than traditional wound cleaning.^48^ In this context, the LbL self-assembly technique can be used to create antibacterial PEs multilayered coatings and films, which help control bacterial growth by mitigating bacterial colonisation.^49^ Antibacterial compounds, such as metallic nanoparticles and small biomolecules like manuka honey, can be incorporated into the multilayers to enhance their efficacy. For instance, silver nanoparticles (AgNPs) are widely used as antimicrobial agents due to their ability to inhibit the growth of both Gram-positive and Gram-negative bacteria. AgNPs can be incorporated within the PE solution, contributing to electrostatic interaction (positive charge) between the layers, and supporting stability in the LbL assembly.^50^

The use of LbL multilayered coatings in wound care incorporates advanced antibacterial properties to prevent infection while promoting tissue repair. Table 2 summarises some of the recent works on LbL self-assembly that incorporate antibacterial agents, evidencing some of the properties and key outcomes. These coatings employ materials like HA and CHT combined with antibacterial agents such as AgNPs, which are effective against a broad spectrum of bacteria and crucial for preventing infections in skin wounds. Still, some challenges associated with silver nanoparticles include nanotoxicity and complex fabrication processes that may affect the coatings' stability. Moreover, polymers such as amino cellulose (AC), a polycationic antibacterial conjugated with AgNPs, are used to modify nanoparticles to employ antibacterial properties on the surface, providing reactive surface groups for the immobilisation of antibodies specific to *Staphylococcus aureus*.^51^ This modification enhances the bactericidal efficacy of the nanoparticles against the targeted bacterium, resulting in a higher antibacterial effect.^52^

**Table 2:** Summary of selected antibacterial LbL multi-layered coatings and key outcomes, where (+) represents the advantages and (−) the limitations

LbL assembly	Characteristics/Outcomes	Ref
(NP_AC-HA_/HA)_5_	(+) anti-bacterial reduction < 60 min (+) no impact on human cell viability (−) potential toxicity	52
(MH/PAH)_8_	(+) highly exuding wounds (+) bacterial prevention capability (+) 100% viable, healthy fibroblasts	53
(HA/ACAgNPs)_50_ (HA/CHTAgNPs)_50_	(+) efficient antimicrobial agent (both Gram-negative and Gram-positive bacteria) (+) limits the risk of infections in the burn, surgical wound, or injury (−) nanotoxicity (−) complex fabrication and poor colloidal stability	49
(CHT-AgNPs/HEP)_7_ (CHT-AgNPs/HEP)_8_	(+) antibacterial reduction (+) long-term continuous anti-bacterial reduction > 1 month	54 and 55
(HEP/CHT)_5_	(+) provide both skin nano-structural and biochemical support (+) ability to control the structure and composition release time	44
(CHT/HA)_15_	(+) efficient antibacterial properties (+) good mechanical properties (+) coatings degrade within 4 days (+) antifungal properties	16 and 23
(PLL/HA)_5_	(+) controlled thickness (+) wettability based on the pH level (+) native ECM-mimetic system fabrication, cell adhesion	56 and 57
(HEP/COL)_10_	(+) mimic the native extracellular matrix, and provide cell adhesion (+) HEP store and releases growth factors	58

LbL configurations like CHT (polycationic) with HEP (polyanionic) offer antibacterial properties and structural support mimicking the extracellular matrix, the diverse LbL assemblies enable quick antibacterial action (less than 60 minutes in some cases) and sustain viability in human cells without toxicity, still requiring precise control over degradation rates and composition to be effective. To address issues associated with naturally derived polymers, such as their faster rates of biodegradation, synthetic polycationic PEs are being considered for their antibacterial properties and degradation characteristics. For instance, poly(allylamine hydrochloride) (PAH) has been exploited to create nanostructured LbL coatings on biomimetic electrospun poly(ε-caprolactone) meshes, forming alternating layers with Manuka Honey (MH). In the coating process, PAH contributes to the formation of more rigid layers compared to MH, enhancing the structural integrity of the nanostructured meshes.^53^ Similarly, poly-l-lysine (PLL) is another synthetic polycationic polymer known for enhancing cell adhesion, nanoparticle uptake, and biocompatibility in a variety of biomedical applications due to its positively charged surface, which interacts favourably with negatively charged cell membranes and biomolecules.^59^ When using strong polycationic polymers, it is crucial to maintain a careful balance between antimicrobial efficacy and biocompatibility for their clinical application.

### 2.3. Exploiting layer-by-layer assembly in wound healing

Acute and chronic wounds present significant clinical challenges, necessitating advanced wound healing techniques. The LbL self-assembly method provides a versatile approach that targets both infection prevention and tissue regeneration. LbL technique has the potential to transform wound care by customising coatings for different wound types and developing next-generation products for antimicrobial activity and tissue healing. For example, Criado-Gonzalez *et al.*^26^ presented a comprehensive overview of the LbL self-assembly of natural and synthetic PEs in the biomedical field, addressing the impact of various factors on modulating the assembly mechanism of multilayers. These factors include PEs concentration, pH, molar mass, and preparation method. The study highlights the advantages of this method in tuning the biodegradability and biocompatibility of multilayers, effectively mimicking biofunctional properties that promote wound healing.

As previously mentioned, LbL assembly offers significant benefits in wound healing applications due to its simplicity and versatility. This method can be applied to substrates of various shapes and sizes, making it highly adaptable for different wound care scenarios. The ability to fine-tune these properties ensures that the LbL assembly can be effectively exploited to enhance WHP, offering a promising approach for future biomedical applications. Various studies have demonstrated the effective use of LbL assembly in wound healing. Hsu *et al.*^60^ developed a pH-controlled, drug-loaded tuneable mechanism for *in vivo* implantation. They constructed 20 layers of tetra PEs (chitosan and poly(β-l-malic acid) (PMLA)) cross-linked *via* biorthogonal click chemistry of (CHT/poly(*b*-l-malic acid) with azide moiety and (PMLA-az)/lysozyme/poly(*b*-l-malic acid) + dibenzocyclooctyne moiety (PMLA-DBCO))_20_ which extended the tuneable release from three to six days. Similarly, Sousa *et al.*^4^ demonstrated the use of chitosan, alginate, and hyaluronic acid in wound dressing, showing that LbL self-assembly improved cellular adhesion or proliferation, which enhanced the WHP and restored the structural and functional properties of wounded skin. This methodology demonstrates the potential to speed up wound healing while providing a treatment that is non-toxic and non-allergenic. The selected PEs offered capabilities such as exudate absorption, gas permeability, antibacterial properties, and durability in a moist environment.

Table 3 summarises diverse combinations of biopolymers that exhibit unique properties and outcomes critical for enhancing wound recovery. Each layer configuration is tailored to exploit specific biocompatibility, degradation, and functional characteristics that align with the needs of effective wound management. For instance, the manufactured multilayer membranes consist of 100 layers of tetra PEs CHT/ALG/CHT/HA(-DN)_100_ where HA-DN is a modified HA with dopamine (DN). The longevity of these multilayer membranes was investigated both *in vitro* and *in vivo*. The *in vivo* test showed that the membranes maintained good cell viability and activity on the surface for seven days. Although the bilayer composites were reduced on the seventh day, the membrane lasted for up to 21 days in the *in vivo* assay.^54^ Further, three-layer assemblies CHT-MH/PVA/ALG, where polyvinyl alcohol (PVA) enhances the formation of physically cross-linked hydrogels with good mechanical properties, biocompatibility, and controlled release capabilities for wound care applications when combined with natural polymers and loaded with antibacterial agents like Manuka honey.^61^ The multilayer structure demonstrates excellent water vapour transmission, fluid drainage, and antimicrobial activity, which are crucial for maintaining a moist and infection-free wound environment.

**Table 3:** Summary of selected polyelectrolyte multilayers (PEM) for skin repair and key outcomes, where (+) represents the advantages and (−) the limitations

LbL assembly	Characteristics/outcomes	Ref
(CHT/ALG/CHT/HA-DN)_100_	(+) mimic the native extracellular matrix (+) continuous WHP 21+ days	54
CHT-MH)/PVA/ALG	(+) exhibited reasonable water vapour transmission rate (+) excellent light transmittance (+) fluid drainage ability (+) effective antimicrobial activity against	61
(HEP/PEI)_8_	(+) exhibited reasonable water vapour transmission rate, (+) excellent light transmittance (+) fluid drainage ability (+) effective antimicrobial activity against	61
CHT/HA)_7_CHT	(+) increase in the mechanical properties of the film (+) increase in cell adherence and effective gram-positive (−) limited effect towards gram-negative	62
CHT-CS	(+) lasts more than 48 hours (+) high cell density (+) clear porosity (+) the physicochemical properties (+) controlled concentration level	63
(CHT/HA/PCDQ)	(+) antioxidant, antibacterial and anti-inflammatory (+) improvement in the wound healing (+) tissue granulating (+) fibroblast migration (+) 3 days replacement interval with significate healing time on day 18	64
PEI/(HA/COLI)5/HA	(+) natural components from the ECM (+) COLI promoter for cell adhesion (+) thin fibres formation (+/-) must end with HA for better cell adhesion behaviour (−) films are not constituted of homogeneously distributed	65 and 66
(CHT/HA)15	(+) promote cell adhesion (+) cell and human fibroblast growth on implant surfaces	67

The heparin (HEP) and polyethyleneimine (HEP/PEI)_8_ configuration similarly supports optimal wound moisture and antimicrobial efficacy.^61^ However, some combinations, such as (CHT/HA)_7_CHT, while improving mechanical properties and cell adhesion, show limited effects against Gram-negative bacteria, suggesting an area for enhancement.^62^ CHT and chondroitin sulphate (CS) forming CHT-CS maintain high cell density and clear porosity, which indicates its ability to support cellular activities and physicochemical stability in the wound environment.^63^ Other assemblies like CHT, HA, and phosphatidylcholine (PCDQ) forming (CHT/HA/PCDQ) offer benefits, including antioxidant, antibacterial, and anti-inflammatory properties, which are vital for fibroblast migration and tissue granulation.^64^ Nevertheless, the poly-(ethylenimine) (PEI), HA, and COLI forming (COLI)PEI/(HA/COLI)_5_/HA assembly faces challenges with uneven film distribution, which impacts its effectiveness.^65,66^ Each bilayer assembly provides distinct advantages for wound care, though some also present specific limitations that need addressing to optimise healing outcomes.

LbL self-assembly has shown promise in various research studies for wound healing, but clinical evidence supporting its effectiveness in wound injuries is still limited. While different naturally based PEs have demonstrated advantages in wound healing through the LbL assembly, the method has not yet been extensively practised or efficiently investigated for clinical translation. Current limitations include the time required to produce multilayers, consistency in manufacturing uniform and homogenous films, and the labour-intensive nature of the procedure. To address these challenges, a method capable of delivering LbL assembly consistently and reliably in a short time, using off-the-shelf PE, is needed. This method should effectively provide the required physicochemical and biological properties.

**Fig. 3:**
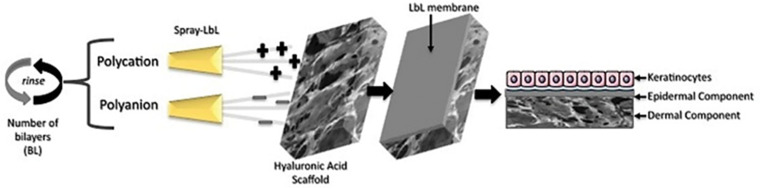
Schematic approach of spray‐assisted layer‐by‐layer assembly on hyaluronic acid scaffolds for skin tissue engineering. Keratinocytes are seeded on top of the membrane, forming a cell monolayer. The LbL membrane acts as an epidermal substitute, which adheres to the dermal component (the porous hyaluronic acid scaffold). Reproduced from ref. 12 with permission from John Wiley and Sons, Journal of Biomedical Materials Research Part A, copyright 2023.^12^

The LbL spraying technique emerges as a powerful, effective, and scalable coating method for depositing and tailoring PEM films with various functional features to promote tissue regeneration. The spray-assisted LbL assembly technique has been exploited in tissue engineering approaches, such as the development of a single epidermal-dermal scaffold to treat full-thickness skin defects (Fig. 3). This technique allows for the rapid and controlled depositing of polyelectrolytes, such as hyaluronic acid and poly-l-lysine, achieving up to 150 bilayers quickly and efficiently.^68^ In general, the use of functional medical devices for wound healing has the potential to minimise the admission demand of accident and emergency departments by providing timely, effective and more accessible treatments. Due to the current limitation, new technological approaches have been proposed in the last few decades for facilitating skin tissue regeneration.

## 3. Bio-based LbL assembly and medical technologies for skin repair

**Fig. 4:**
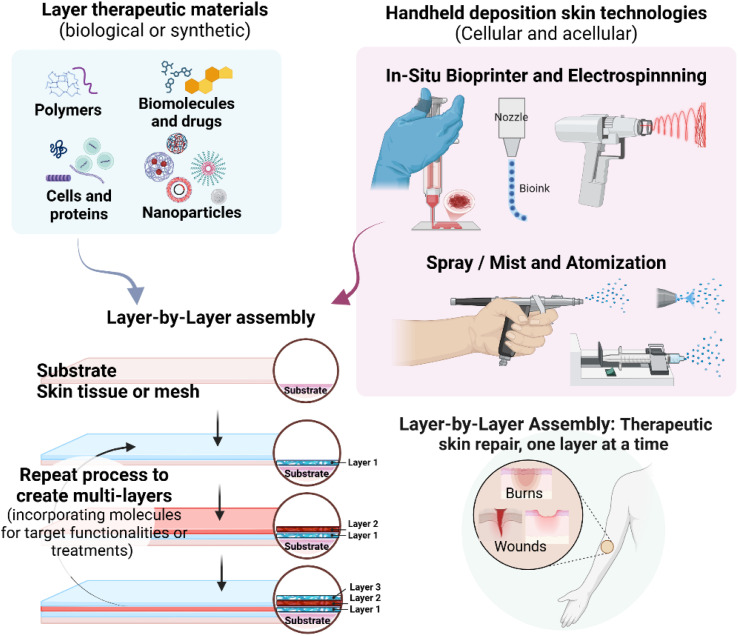
Schematic overview of the therapeutic skin Layer-by-layer assembly onto skin tissue or meshes as substrates to deposit an extensive choice of therapeutic materials using handheld technologies to treat the wound and assist in the wound healing process for skin burns and trauma injuries. Created in https://BioRender.com.

The range of materials for forming multilayers in skin regeneration is expanding with new assembly technologies. As LbL assembly techniques grow, the boundaries of what constitutes LbL assembly blur. Weak interactions like van der Waals forces and biological recognition have extended the scope of LbL assembly, allowing macromolecules to arrange into stable conformations. This section highlights innovations in skin treatments using LbL approaches, showcasing unconventional methods (*e.g.* spray, *in situ* bioprinting and electrospinning) and combinations of building blocks (*e.g.*, cells, diverse materials, and polyelectrolytes). The potential and applicability of these technological methods in combination with therapeutic polyelectrolytes, materials and molecules are also illustrated in Fig. 4.

LbL encapsulation of living cells and microorganisms is promising for engineering cellular therapeutic approaches with enhanced properties. Applications include encapsulation and functionalisation of mammalian cells and thick tissue formation.^24^ Spatially organised films can fabricate complex systems mirroring natural complexity. However, the cytotoxicity of conventional LbL materials and cationic molecules requires careful study,^69^ such as high concentrations of polycations (*e.g.*, PEI, PLL), can limit their biomedical applicability.

Stacking cells in an LbL fashion has inspired tissue engineering to move from scaffold-based approaches to scaffold-free constructs and organ printing, promising for drug screening, toxicological testing, and precision medicine.^70,71^ For instance, Akashi group developed 3D tissues by coating cells with LbL films made of ECM proteins.^72^ Initially, fibroblast layers were coated with fibronectin and gelatin (FN/G) films before adding more cells. Cells maintain a negative electrostatic charge.^73,74^, aiding in LbL assembly. This evolved into single-cell LbL coating, called the cell-accumulation technique, allowing further vascularisation with HUVECs.^75^ Mouse fibroblasts embedded in FN/G coatings proliferated, increasing tissue thickness from 10 µm to 50 µm over four weeks. Heterocellular tissue constructs can be prepared within 2–3 days, with up to 8–10 layers, limited by cell-seeding density and nutrient supply. Therefore, biological constructs' properties can be tailored by choosing different cell layers, mimicking the complexity of actual tissues.

### 3.1. Spray-assisted layer-by-layer innovations for skin repair

Pleguezuelos-Beltrán *et al.*^43^ investigated worldwide clinical trials for skin therapeutic devices up to May 2021 using https://www.clinicaltrials.gov/, focusing on skin sprays for treating various skin conditions. The study identified 104 clinical trials, psoriasis had the most trials (45), followed by chronic wounds, burns, dermatitis, and other conditions. Skin sprays were sprays that often-contained autologous cells, while acellular sprays included antiseptic solutions, anti-inflammatory drugs, and polymeric biomaterials. For instance, corticosteroids were commonly used for psoriasis, with Clobex® Spray being the most prescribed. Other notable products were AOBiome's B244 for the skin microbiome. Noveome's ST266 for burns and dermatitis targets tissue repair by delivering multiple growth factors. Such specialised formulations underscore the versatility of spray devices and their ability to deliver targeted therapies to skin conditions in a convenient, minimally invasive manner. In addition, the study “Safety of Gebauer's Pain Ease and Gebauer's Ethyl Chloride” (NCT04207710) assessed whether these sprays increased microbial growth on the skin when used as numbing agents. Henry Ford Health System concluded that there was no significant impact on skin sterility and that the sprays did not promote bacterial growth.

The discovery of autologous cell suspension, CellSpray®, in 1994 marked a significant advancement in the field. This method facilitates the aerosol delivery of cells to debrided burn and donor skin graft areas, optimising cell distribution to the wounded surface and enhancing the overall wound healing process.^76^ Veazey *et al.*^77^ examined the cell survival rate and viability of dermal fibroblasts using an airbrush, assessing various nozzle diameters (312, 484, and 746 µm) and air pressures (41, 55, 69, 96, and 124 kPa) settings. The study found that a larger nozzle diameter and lower air pressure resulted in higher cell survival rates. While higher air pressure could effectively deliver the stem cells, it also caused shear stress, reducing short-term survival rates. Therefore, spray devices intended to spray cellular suspension and to incorporate PEs must be evaluated for pressure and nozzle diameter, as the viscosities of the components may change. Controlling air spray pressure is crucial for delivering a reliable treatment. Cohen *et al.*^78^ introduced an aerosol device containing three compressed air injectors for specific vials, two injectors to spray the sterile tissues adherence, and a third one to spray an epidermal cell suspension. This device showed promising recovery with 94.2% epithelialisation, though gravitational forces caused irregular coating, affecting homogeneity.

Multiple parameters associated with spraying devices in the medical field warrant consideration and enhancement for future technological advancements. These parameters include the pressure and spraying mechanism, fluid viscosity, spraying distance, and angle, all of which may significantly influence the WHP. The relationship between the pressure exerted by the spraying pump and the cell survival rate depends on the level of pressure, where higher levels can lead to a decrease in cell viability.^79^ The materials or fluid's viscoelastic properties are controlled by multiple factors based on molecular weight,^80^ temperature and concentration, which all have a direct effect on the entire mechanism.^81^

Cell viability after spray application is a critical consideration due to the mechanical stresses imposed during the process, which can lead to cell damage, such as membrane elongation and decreased viability. Still, factors like nozzle diameter, material viscosity, and delivery velocity, microenvironment, significantly affect these shear stresses.^82^ Research using saline carriers has shown a high survival rate of over 90% when using low air pressure and large nozzle sizes.^77^ The survival rates of cells are achievable with comparable nozzle sizes and air pressures. It is found that the metabolic activity of sprayed cells remains stable, indicating that cell viability is maintained post-spray, and the proliferation of these cells increases over time.^83^ This underscores the need for careful modulation of the applied force to preserve the integrity and functionality of the cells during spray-based applications.^84^

Moreover, spray distance is a crucial parameter affecting the dispersion of particles, the effectiveness of the procedure, and the coverage area. Due to the different types of spraying mechanisms and the most common, using an airbrush mixing of two fluids (air and liquid), a minimum distance of 10 cm is a safety measure to prevent the risk of air embolus in topical and endoscopic surgery applications.^83^ Spraying angle presents partial parameter limitation with two direct factors, device and procedure orientation, which lead to fluid run-off effect based on application approach, based on biomaterials and concentrations.^85^

So far, there is no medical device aimed at applying multi-layered coatings or therapeutic LbL films to promote wound healing. Thus, this review summarises a range of different devices that could support the build-up of multi-layered coating and/or application of therapeutic PE, and/or provide *in situ* wound dressing substrates for further LbL assembly. Their technological challenges and opportunities for skin repair are presented below. In addition, different patented technologies for wound healing (including patent phase and U.S. FDA-approved devices^33^) are summarised in Table 4, showing treatment suitability, requirements, and efficiency.

**Table 4:** Handheld machines rely on regenerative medicine such as stem cells, skin grafts, and biomaterials along with other techniques to treat the wound and assist in the wound healing process for skin burn and trauma injuries

Device name (manufacturer)	User and delivery preparation	Required time	Wound coverage	Cells survival rate	Patent/FDA 510(K) device number and clinical trial status	Ref
**Regenerative approaches**						
Auto micro atomization delivery (AMAD)	User: healthcare professional c preparation: 1. Stem cells isolated from a skin sample 2. Prepare the stem cell solution and placed it in a reservoir 3. Spray (tips 200 µm) stem cells (3–18 cm)	40–60 min	7–10 days of full wound closure of 1 cm × 1 cm	94.8% < *x* < 95.2%	(CN106620974A) clinical trial: unknown	86
SkinGun (RenovaCare)	User: healthcare professional^c^ preparation: 1. Stem cells isolated from a skin sample 2. Infuse skin grafts with enzymes to create solution stem cells. 3. Spray cells with disposable syringe	90–120+ min	Doner area 50 cm^2^ ∼Burned area 2000 cm^2^^a^	97.3%	(KR20190042547A) (US10376658(Clinical trial: NCT04890574 on burns, burns deep second degree	89 and 92
Cell spraying device, method & sprayed cell suspension (RenovaCare-cell mist) initial model	User: healthcare professional^c^ preparation: 1. Stem cells isolated from a skin sample 2. Infuse skin grafts with enzymes to create solution stem cells. 3. Spray stem cells	90–120+ min	Doner area 50 cm^2^ ∼Burned area 2000 cm^2^^a^	72.7% < *x* < 91%	(US20170304600A1) clinical trial: NCT04890574 on burns, burns deep second degree	93–95
RECELL system (Formerly spray-on-Avita medical) b	User: healthcare professional preparation: 1. Incubate a small sample of skin 2. Test and rinse scrape for disaggregation 3. Spray the filtered cell suspension three simple steps: skin graft; emerge skin graft with a proprietary enzymatic solution; spray cells with the suspension device	30–120 min+	4 cm^2^ DSST area relative to 320 cm^2^ burnt area	> 95%	US10631974) clinical trials: multiple NCT02994654, NCT01138917, NCT02380612, NCT03333941, NCT03626701 on burns; NCT05386368 on photoaging and carbon dioxide laser; NCT01743053 on Venous Leg Ulcers	89, 96 and 97
Handheld skin bioprinter	User: healthcare professionals^c^ preparation: 1. Prepare bioink (premixed biomaterials and cells) and cross-linker solutions 2. Hold the instrument to deposition on wound 3. Bioink delivery *in vitro* or *in vivo* (3 ml bioink covers up ∼100 cm^2^ in 0.8–2.1 min)	0.8–2.1 min per wound size 100 cm^2^ per 3 ml of bioink solution	3 ml of bioink for 100 cm^2^	¼ fully healed wound	(WO2018064778A1) clinical trial: unknown	1 and 88
*In situ* bioprinting (delivery system)	User: healthcare professional^c^ preparation: 1. Prepare the bioink and cells (2 cartridges with different payloads 2. Place the patient at the machine for *in situ* tissue bioprinting (XYZ nozzle diameter 8 × 260 µm with 100 µm precision) 3. Operate on injury, robotically scanning wound (movement with the assistance of laser scanner to cover wound bed system)	120+ min	Not detailed	> 95% wound closure week 5	(US20200238024A1) (EP2683328B1) clinical trial: Unknown	98
Spincare (SPINNET system)	User: Healthcare professional^c^ preparation: 1. a syringe is placed inside the device and adjusted using a liner-pressing mechanism 2. Place the device at 20 cm (electrode: Must be fixed at the patient's body-electromagnetic field enhancement) 3. Spray directly on a wound (personalised stem cells in a disposable syringe)	Based on treatment	Customized to required coverage. Excessive nanofibrous dressing (PND) does not affect the procedure	—	(US20170239094A1) clinical trial: NCT02997592 on partial skin burns, NCT05944250 on recessive dystrophic epidermolysis Bullosa	48 and 99

**Acellular approaches**						
UltraMist therapy (The MIST™ therapy-Sanuwave)	User: healthcare professional^c^ preparation: 1. Prepare the saline 2. Connect the cannula to the ultrasound nozzle 3. Setup the generator to the required frequency (40 kHz) to alter the saline to mist spray (spray distance of 10 mm)	3–20 min based on area and operative team for 3 times per week	Reduction varies based on patient response to treatment (mean 85.2% reduction in 7 weeks)	—	(US6569099B1) clinical trial: NCT06813430 on wound healing, non-contact low frequency ultrasound, Lalonde protocol, Fingertip Amputation	100–102

a Approximately for every donor site skin tissue area (DSST) starting with 50 cm^2^ (50+) can cover the burned area 2000 cm^2^ (increment of 1+).

b ReCell requires 20 minutes more time than the conventional skin graft method.^91^

c Healthcare professional includes nurses, trained operator, trained technicians, medical doctors, and trained personnel.

#### 3.1.1 AMAD device

Chang *et al.*^86^ Introduced the AMAD device, a novel compact system that assists with wound injury from substantial fluid loss. This therapeutic cell-delivery system provides a 45% even droplet size, compared to other systems that offer uneven spraying amounts, limit cell uniformity, and reduce cell survival rate.^87^ Tested with cellular suspension, it addressed critical designing factors such as density, viscosity and spraying distance, domain, and angle. It was noted that the viscosity influenced the spraying angle and droplet distribution.

#### 3.1.2 Spray-on skin device

Spray-On Skin (Avita Medical) and SkinGun (RenovaCare) were initially developed to treat burn injuries; however, their current application has expanded to include cosmetic uses.^88,89^ Esteban-Vives *et al.*^90^ combined the RenovaCare Skin-Gun with a smaller orifice to discharge the jet stream of a 10 ml syringe containing cell solution. The alternating movement spray technique was used to increase the maximum coverage area when sprayed at 20 cm. The study focused on the homogeneity of spray coverage at the wound site and the implantation of the spraying mechanism to maximise coverage area. The aerosol method of cell suspension was cost-effective, less invasive, and efficient,^87^ leading to better wound coverage and decreased healing time.^89^ Furthermore, RenovaCare and AMAD systems share the characteristics of an airbrush mechanism. The spray quantity can control over spray quantity through a thin rod and pressing mechanism, which determines the relative quantities of both air and solution depending on the spraying distance.

#### 3.1.3 ReCell device

The ReCell system employs a simple approach to a complex procedure, particularly in delivery preparation. Traditional skin grafting techniques offer a more rapid application compared to the ReCell system, evidencing enhanced efficacy. The ReCell system requires a smaller skin donor area to treat wounds, making it less invasive and providing an 80-fold cell expansion.^91^ This device, previously used to spray enzymatically digested tissues, has the potential to spray therapeutic naturally derived PEs such as collagen, hyaluronic acid, polysaccharides, and cellular suspensions.

#### 3.1.4 MIST™ device (acellular)

The MIST™ therapy system uses an ultrasound device that does not contact wound injuries. It employs a piezoelectric transducer horn to convert electrical energy to mechanical energy, creating an acoustic pressure output.^103^ Used with saline solution, this device could offer the opportunity to spray low-viscosity PEs. Kataoka *et al.*^104^ noted the lack of evidence on the efficacy of non-contact low-frequency ultrasound (NLFU) in accelerating wound healing, suggesting the need for further studies. While evidence shows that the device promotes wound healing and reduces complicated wounds, the procedure requires at least three follow-up visits per week.^101^

### 3.2. Bioprinting-based technologies

Bioprinting technologies enable the deposition of hydrogels and crosslinked solutions directly onto the skin or *in vitro* to create skin constructs in a layer-by-layer approach. Although this process does not inherently involve layer-by-layer assembly through electrostatic interactions, these technologies can be adapted to process viscous PEs materials into hydrogel substrates or wound dressings. This approach allows for skin regeneration, with *in situ* crosslinking to control the thickness and composition of the layers within the construct.

#### 3.2.1 Handheld skin bioprinter

An innovative device designed for the *in situ* formation of biomaterial and skin tissue sheets directly onto wound sites. Weighing less than 0.8 kg, this compact instrument allows for the conformal deposition of bioinks comprising biomaterials and cells onto target surfaces, effectively accommodating various wound topographies. The device utilises inkjet-based bioprinting technology, coordinating the flow rates of bioink and cross-linker solutions with the movement speed of a pair of rollers to ensure consistent sheet formation.^105^ Studies have demonstrated its compatibility with diverse bioinks, including those containing dermal and epidermal cells within ionically cross-linkable biomaterials like alginate and enzymatically cross-linkable proteins such as fibrin. The rapid cross-linking facilitated by the device results in uniform thickness and composition of the biomaterial and cell-laden sheets. Preclinical models indicate that the handheld skin bioprinter effectively covers wounds, including inclined surfaces, enhancing wound healing and tissue regeneration. Its portable design enhances accessibility in various clinical settings, from operating rooms to emergency care. Despite challenges in optimising bioink formulations and ensuring cell viability during printing, this bioprinter shows an advancement in regenerative medicine, promising a minimally invasive and efficient approach to skin regeneration.^106,107^

#### 3.2.2 *In situ* skin tissue 3D bioprinting

Shpichka^108^ reported the benefit of using the 3D bioprinter and inkjet methods, which ensure full-thickness tissue restoration followed by vasculogenesis due to progenitor cell migration and angiogenesis. However, most *in situ* 3D bioprinting machines rely on cross-linking of biopolymers for skin substitution, posing challenges for extensively customised skin substitutes. Innovative methods like an *in situ* skin bioprinting system and a handheld skin bioprinter offer better accessibility and flexibility for effective wound coverage,^98,109^ addressing issues related to wound size, operation duration, and preparational time.

### 3.3. *In situ* LbL electrospun-assisted wound dressing

The Spincare system focuses on electrospun healing fibres (EHF) to advance wound care and embeds cells for tissue regeneration. The EHF matrix covers wounds using electrospun polymer nanofibrous dressing (PND), allowing precise and targeted application. Therefore, this technique is proposed to be advantageous for creating a wound dressing and substrate for a therapeutic layer-by-layer approach exploiting therapeutic PEs and antibacterial molecules. The non-contact treatment method utilised by the Spincare system requires further investigation, such as excessive exudation, tearing of the dressing, difficulties in adhesion removal, and issues related to aligning the electrospun fibres with the patient's electromagnetic field attachment for optimal adherence and effectiveness. To facilitate the effective deposition of electrospun fibres by the Spincare system, it is essential to establish a grounded electromagnetic field attachment for the patient, which serves as a target object, ensuring that the fibres are precisely directed and securely attached to the wound surface.^48^

## 4. Concluding remarks and future perspectives

Skin wound management is a significant global healthcare challenge, with high costs, substantial morbidity, and extended recovery times. LbL technology offers a transformative, versatile, and scalable solution for advanced wound care by enabling precise deposition of bioactive compounds and mimicking the native extracellular matrix. The use of natural and synthetic polyelectrolytes (PEs) creates a biocompatible environment that accelerates wound healing, offering benefits like antimicrobial properties, moisture retention, and structural integrity for cell adhesion and proliferation. The controlled release capability of LbL coatings supports sustained therapeutic delivery, improving patient outcomes and minimising repeated interventions. Smart materials that respond to environmental cues (*e.g.*, pH, temperature) could optimise treatment outcomes and personalise wound care. LbL coatings can incorporate therapeutic agents such as growth factors, antimicrobials (*e.g.*, silver nanoparticles), and bioactive compounds (*e.g.*, methylglyoxal from Manuka honey), enabling targeted drug delivery and enhanced efficacy. This flexibility addresses various wound types, from acute to chronic wounds. However, challenges like manufacturing uniformity, layer deposition inconsistencies, and reliability of LbL coatings need to be addressed for widespread clinical adoption.

Innovations in spray-assisted technologies, such as the SkinGun and ReCell, have optimised spray parameters to improve cell viability and wound coverage, leading to increased cell survival rates and reduced wound closure time. These advancements contribute to decreased morbidity, mortality, hospital stays, and healthcare costs. Despite the success of Spray-on Skin technologies, techniques like cell suspension and tissue-engineered skin substitution face limited adoption due to cell expansion time, wound size considerations, and high operational costs.^1,43^ Regulatory approval processes also present substantial barriers, highlighting the need to streamline pathways and standardise protocols for LbL fabrication and testing.

Future research should focus on developing user-friendly multilayered coating devices to translate versatile, multifunctional LbL assembly approaches into diverse clinical settings, including emergency care and remote healthcare facilities. LbL technology's ability to mimic native skin composites, deliver therapeutic agents precisely, and incorporate antimicrobial properties makes it crucial for complex wound management. Procedure times remain considerable (*e.g.* ranging from 65 to 120 minutes for mid-dermal to full-thickness wounds), straining clinical resources. Integrating handheld, spray-assisted devices capable of rapid, on-demand multilayer deposition could reduce procedure times, lower operational costs, and minimise the need for extensive surgical infrastructure. Successful integration into clinical workflows will require focused research, regulatory alignment, and extensive clinical validation.

To fully realise the potential of LbL assembly for skin repair in clinics, efforts must focus on scaling production, standardising protocols, and bridging regulatory gaps. Strategic multidisciplinary collaborations between (but not limited to) materials scientists, engineers, clinicians, and regulatory bodies will be crucial. Continued innovation and a focus on cost reduction and sustainability could redefine wound care, reduce healthcare costs, and improve patient outcomes globally. Looking ahead, the development of materials compatible with LbL assembly technologies will be essential. These materials must be biocompatible, non-toxic, and capable of forming stable multilayers. Innovations in smart materials that respond to environmental cues (*e.g.*, pH, temperature) will enhance therapeutic delivery and personalization of wound care. By addressing these requirements, LbL-based treatments can potentially achieve widespread clinical adoption and significantly impact global healthcare.

## Data availability

No primary research results, software or code have been included and no new data were generated or analysed as part of this review.

## Conflicts of interest

There are no conflicts to declare.
